# Impact of in-hospital birth weight loss on short and medium term breastfeeding outcomes

**DOI:** 10.1186/s13006-018-0169-6

**Published:** 2018-07-03

**Authors:** Sergio Verd, Diego de Sotto, Consuelo Fernández, Antonio Gutiérrez

**Affiliations:** 1 Department of Primary Care, Balearic Health Authority, 07003 Palma de Mallorca, Spain; 2 Endocrinology Unit. Department of Paediatrics, Son Espases University Hospital, Valldemossa Road, 79, 07010 Palma de Mallorca, Spain; 3 Molecular Biology Unit, Division of Hematology, Son Espases University Hospital, Valldemossa Road, 79, 07010 Palma de Mallorca, Spain

**Keywords:** Breastfeeding, Newborn infant, Birthweight, Human milk, Lactation, Weight losses, Weight gain

## Abstract

**Background:**

The definition for lower limit of safe birthweight loss among exclusively breastfed neonates is arbitrary. Despite this, in cases of great in-hospital weight loss, breastfeeding adequacy is immediately questioned. The aim of this study was to examine the relationship between weight loss at discharge from hospital, when babies are ready to go home, and eventual cessation of exclusive breastfeeding since birth.

**Methods:**

This is a secondary analysis of a cohort study. Study participants were 788 full term, breastfed and stable babies, born in 2007–2012 consecutively enrolled to primary care pediatric clinics in Majorca, Spain. Data were collected by chart review. The main predictor was birthweight loss at discharge. Extreme weight loss was defined as the 90th and 95th centiles of birthweight loss for babies who were delivered by vaginal delivery and by cesarean section. Main outcomes were cessation of exclusive breastfeeding by 7, 15, 30 and 100 days of life. Multivariate regression analysis was performed to study the relationship of selected variables with exclusive breastfeeding cessation since birth.

**Results:**

We observed a median weight loss of 6%. In bivariate analysis, quartiles of birthweight loss at discharge were predictive of exclusive breastfeeding cessation at 15, 30 and 100 days postpartum. In multivariate analysis: in-hospital weight loss above the median did predict exclusive breastfeeding cessation by 15, 30 and 100 days of life, Adjusted Odds Ratios (AORs) (95% Confidence Intervals [CIs]): 1.57 (1.12, 2.19), 1.73 (1.26, 2.38) and 1.69 (1.25, 2.29), respectively. In contrast, we did not find that newborn extreme weight losses were associated with exclusive breastfeeding cessation.

**Conclusions:**

We report that extreme birthweight loss does not trigger immediate formula supplementation. We do not identify any cut-off values to be used as predictors for the initiation of supplementary feeding, this research question remains unanswered.

## Background

Almost all newborns lose weight from birth to discharge [1]. During the first days of life, exclusively breastfed neonates lose around 6% of their birthweight prior to beginning consistent weight gain [2, 3]. Based on a simplistic view, measuring newborn weight change would portend insufficient milk supply. However, assessment of effective breastfeeding must be founded on more than early weight loss in order to avoid the well-known association of pronounced birthweight loss and exclusive breastfeeding cessation [4]. This subject has a lot of interest because one needs to move away from the danger zone of insufficient mother’s milk intake without escalating towards the danger zone of discouraging breastfeeding. Dehydration and hyperbilirubinemia are more common in breastfed infants. Consequently, weight monitoring has been used to assess the adequacy of breastfeeding and the need for formula supplementation, which prompts breastfeeding cessation [5–7]. In some cases, it is not easy to protect babies from breastfeeding cessation and at the same time from suboptimal breastfeeding complications.

Lactation failure, as well as remedies for this condition are known since the *Papyrus Ebers* (Egypt, 1550 BC) but current knowledge cannot support that any pronounced birthweight loss points at breastfeeding failure [8]. Weight charts to identify infants who need breastfeeding support have been recently developed [9]. However, they cannot be used to project individual weight changes in neonates. For this reason, Wilbaux et al. [10] have developed a model that can predict individual weight change during the first week of life. Unfortunately, no one approach sets the birthweight change at which intervention should be initiated to prevent clinical complications. The lack of evidence to estimate an excessive weight loss does not assist neonatal practitioners in combining breastfeeding promotion with the prevention of pathological birthweight loss [11]. To keep on exclusive breastfeeding in the setting of adequate weight gain for a number of weeks is an obvious indicator of breastfeeding adequacy, but previous research has scarcely studied breastfeeding outcomes beyond hospital discharge on babies that have suffered substantial birthweight loss [4, 12]. Most papers have focused in describing risk factors for birthweight loss [13, 14] or in categorizing birthweight loss immediately after birth [15–17]. No doubt further research is needed in this area with special attention to the effects of birthweight loss on breastfeeding trajectories. In an attempt to help disentangle what is physiological birthweight loss from what predicts breastfeeding failure, we have undertaken a secondary analysis of data collected for gestational diabetes research [18] to explore the association between birthweight loss and exclusive breastfeeding. This study describes how different degrees of birthweight loss predicts giving up exclusive breastfeeding in the short and in the medium term among breastfed term neonates cared for in a setting devoted to supporting breastfeeding.

## Methods

### Selection criteria and setting

We explored the relationship of common clinical variables and exclusive breastfeeding recorded during the hospital stay with cessation of exclusive breastfeeding from day one until day 100 of life. We enrolled a convenience sample of 788 stable infants between 37 and 42 weeks of gestation. After birth, mothers were encouraged on demand breastfeeding. Infant feeding policies at the birth center and pediatric centers were based on the World Health Organization’s Ten Steps to Successful Breastfeeding standards [19].

Newborn care nowadays differs place to place. In our nursery, the usual length of stay is 48 h after a vaginal delivery and 72–96 h after a caesarean section; mother and baby stay in the same room 24 h a day and partners are welcome to offer support to new mothers. Families receive basic newborn care information and are told about breastfeeding support available near them. They are encouraged to seek neonatal health care in the first week after birth.

The enrollment phase lasted from January 2007 to December 2012. Breastfeeding mothers attending four general care pediatric clinics in a middle-class neighborhood in Majorca, Spain, were invited to participate in “a study on infant feeding” upon their first well-child visit.

### Data measures

Based on 1-h 50-g-oral glucose tolerance test (1-hOGTT) and 3-h 100-g-oral glucose tolerance test (3-hOGTT), pregnant women were stratified into the following three glucose tolerance groups: normal glucose tolerance, defined by normal 1-hOGTT results (1-h plasma glucose < 7.8 mmol/L); mild impairment of glucose tolerance, defined by a single abnormal value ≥7.8 mmol/L but < 10.6 mmol/L; or gestational diabetes, defined by at least two of the following on the 3-hOGTT: fasting glucose 5.8 mmol/L, 1-h glucose 10.6 mmol/L, 2-h glucose 9.2 mmol/L, or 3-h glucose 8.1 mmol/L [17].

Extreme weight loss was defined as the 90th and 95th centiles of birthweight loss for babies who were delivered by vaginal delivery (VD) and by cesarean section. We obtained cut-offs values 10.5 and 11.5% for VD, and 11.5 and 12% for cesarean section.

We defined exclusive breastfeeding as nothing by mouth since birth except breast milk and, possibly, medicines or vitamins.

### Data collection

A secondary analysis of data from a study examining breastfeeding outcomes of mild gestational hyperglycemia was completed [18]. This research was not primarily intended to study breastfeeding variations after birthweight loss. The study protocol has previously been described in detail [20]. In brief, the study was conducted as a review of medical records from a Pediatric office where circa 120 babies are enrolled every year. We extracted additional data on patient characteristics and on infant feeding from the perinatal and neonatal medical records. Variables studied were: maternal age, parity, gestational weight gain, gestational glucose tolerance, type of delivery, birthweight, birth head circumference, gender, type of feeding, admission to the neonatal ward and length of hospital stay. With regard to weight, we considered: weight at birth and weight at hospital discharge. Every day between birth and discharge, all infants were weighed naked with an electronic scale, by a nurse. Information about the duration of exclusive breastfeeding was obtained from mother’s report of how the baby was being fed at the time of scheduled well-baby visits at 7, 15, 30 and 100 days of age.

### Data analysis

Our primary predictor of exclusive breastfeeding discontinuation was birthweight loss. Dichotomization: this variable has been split at the median to form high and low birthweight loss groups. In addition, we carried out a bivariate analysis on exclusive breastfeeding outcomes by day 100 of life after extreme weight loss.

Univariate differences across the groups were assessed using Mann-Whitney test for continuous variables and either chi square or Fisher’s exact test for categorical variables. Multiple regression analysis was used to identify demographic or clinical factors that independently predicted short duration of exclusive breastfeeding. Covariates considered included parity, gender, glucose tolerance status in pregnancy, delivery type, birthweight and neonatal weight loss at discharge. A series of models were constructed using these covariates. The final models included main effects significant at *p* <  0.05. Data were analyzed using the IBM-SPSS (V22.0) Package.

## Results

A total of 788 full term, breastfed babies were included in this study. We have divided participants into two groups (≤ median weight loss and > median weight loss). Demographic and selected clinical data of our study population are summarized in Table 1. This study involved 508 primiparous mother-neonate pairs and 280 multiparous mother-neonate pairs. With regard to other perinatal factors, median gestational weight gain was 12 kg, median gestational age was 40 weeks, and median birthweight was 3260 g in case of weight loss at discharge ≤ median and 3310 g in case of weight loss at discharge > median. One hundred and fifty-four women underwent a cesarean section (19.5% of the total sample). Blood glucose was measured in 788 pregnant women, mildly impaired glucose tolerance was detected in 154 (19.5% of the population). In our infants, admission to the special care nursery was a rare event: it was reported in 28/788 infants (3.5%).

**Table 1:** Baseline participant characteristics by study group

Variable	Neonatal weight loss at discharge ≤ median	Neonatal weight loss at discharge > median	*p* value
	*n* = 403	*n* = 385	
Maternal age, years	33 (18–45)	33 (21–45)	0.97
Parity:			
1	250 (62)	258 (67)	0.13
> 1	153 (38)	127 (33)	
Mildly impaired glucose tolerance	73 (18)	81 (21)	0.37
Gestational weight gain, kg	12 (4–39)	12 (1–30)	0.12
Delivery type:			
Non-assisted	206 (51)	215 (56)	0.21
Instrumental	60 (15)	62 (16)	
Cesarean section	137 (34)	108 (28)	
Weeks of gestation	40 (37–42)	40 (37–42)	0.092
Gender:			
Male	197 (49)	189 (49)	1
Female	206 (51)	196 (51)	
Birthweight, grams	3260 (1995-4390)	3310 (2270-4800)	0.005
Birth head circumference, cm	34 (31–37)	34.5 (31–37)	0.13
Admission to neonatal ward	16 (4)	12 (3)	0.57

Unless indicated otherwise, data are expressed as *n* (%) or median (range)

The degree of initial birthweight loss was predicted by birthweight, with infants with low birthweights showing little weight loss. No significant statistical differences were found between high and low birthweight loss groups when it came to maternal age, gestational weight gain, gestational glucose tolerance, weeks of gestation, parity, delivery type, gender, head circumference at birth or admission to the neonatal ward (Table 1). birthweight loss > median predicted cessation of exclusive breastfeeding by day 100. Weight change data at discharge are summarized using interquartile range in Table 2. We observed a median birthweight loss of 6%.

**Table 2:** Approximate interquartile distribution of the newborn infants according to their weight change at discharge

Newborn weight change at discharge (%)	*n* (%)
≥ −4	210 (27%)
[−4; −6]	193 (24%)
[−6; −8]	220 (28%)
≤ −8	165 (21%)

Using bivariate analyses, birt weight loss quartiles at discharge were not predictive of exclusive breastfeeding cessation at 7 days postpartum. In contrast, at 15, 30 and 100 days, birthweight loss quartiles at discharge were correlated to exclusive breastfeeding cessation (see Table 3). In addition, this considerable significance was sustained after adjustment for gender, parity, impaired gestational glucose tolerance, delivery by cesarean section and median birthweight (Table 4). The model obtained by multiple regression analysis shows that in-hospital > median birthweight loss did not predict exclusive breastfeeding cessation by day of life 7, but it did predict exclusive breastfeeding cessation by days of life 15, 30 and 100. There is no relevant change in the adjusted ORs and in the confidence intervals on days 15 or 30 with respect to day 100. The difference between the two groups of birthweight loss persisted at the more extreme end of exclusive breastfeeding duration.

**Table 3:** Exclusive breastfeeding discontinuation according to newborn weight change at discharge

[Newborn weight change at discharge] *n*	Exclusive breastfeeding discontinuation ≤ 7 days	*p*	Exclusive breastfeeding discontinuation ≤15 days	*p*	Exclusive breastfeeding discontinuation ≤30 days	*p*	Exclusive breastfeeding discontinuation ≤100 days	*P*
[≥ −4] 210	165 (21)	0.15	205 (26)	0.016	229 (29)	< 0.001	339 (43)	< 0.001
[−4; −6] 193	150 (19)		173 (22)		221 (28)		347 (44)	
[−6; −8] 220	150 (19)		229 (29)		276 (35)		394 (50)	
[≤ −8] 165	213 (27)		292 (37)		378 (48)		504 (64)	

Unless indicated otherwise, data are expressed as *n* (%)

Differences across the groups were assessed using Mann-Whitney test

**Table 4:** Adjusted Odds Ratios for the multivariate model predicting exclusive breastfeeding cessation by median birthweight loss

Variable	EBF < vs ≥ 7 days	EBF < vs ≥ 15 days	EBF < vs ≥ 30 days	EBF < vs ≥ 100 days
	AOR (95% CI)	AOR (95% CI)	AOR (95% CI)	AOR (95% CI)
Gender:	–	–	–	1.72 (1.27,2.32)
Male				
Female				
Parity > 1:	0.52 (0.34, 0.8)	0.56 (0.38, 0.81)	0.52 (0.37, 0.74)	0.55 (0.4, 0.75)
Impaired glucose tolerance^a^	–	–	–	1.65 (1.12, 2.42)
C-section delivery:	1.75 (1.19, 2.58)	1.54 (1.08, 2.19)	1.38 (0.98, 1.95)	–
> Median birthweight (range)	–	–	–	0.69 (0.51, 0.93)
In-hospital > Median BWL (range)	–	1.57 (1.12, 2.19)	1.73 (1.26, 2.38)	1.69 (1.25, 2.29)

Abbreviations: *AOR* adjusted odd ratio, *BWL* birthweight loss, *CI* confidence interval, *EBF* exclusive breastfeeding

^a^Pregnant women with an elevated 1-h glucose challenge test but that still do not qualify for gestational diabetes

Considering the possibility that newborn extreme weight loss may be the trigger for formula supplementation and therefore compromise milk supply, we carried out a bivariate analysis with the same variables as in Table 3, but restricted extreme weight loss and 100 days postpartum. Extreme weight loss was not associated with exclusive breastfeeding cessation by day 100.

## Discussion

Our main findings are that our infants were equally likely to be exclusively breastfed by day 100 whether or not they reached extreme weight loss, but we show that newborns whose birthweight loss at discharge from the maternity ward was below the median were more likely to discontinue exclusive breastfeeding by days 15, 30 or 100 of life, but not by day 7 of life. To the best of our knowledge, this study is the first to examine the link between in-hospital birthweight loss and breastfeeding duration beyond month three of life.

From the physiological standpoint it is unknown whether extreme weight loss results solely from voiding and insensible water loss during those days when milk oral intake is still low or it is due mainly to inadequate intake [21]. However, it is worth remembering that the average amount of breastmilk ingested during the first day of life by full term neonates is 15 mL [22]. In addition, very recent research [4] shows that once newborns started gaining weight, similar patterns of weight gain emerged between the group above and the group below the threshold for extreme weight loss. Moreover, not only delayed onset of lactogenesis, but also intrapartum fluid net balance and infants’ rooting movements on day two of life may be involved in food intake regulation and predict birthweight loss [15, 16, 23]. Taken together, current data do not support hospital routines that are associated with both spurious increase in birthweight loss and poor breastfeeding outcomes of extreme weight loss infants. Depending on what kind of feeding directions are provided to mothers when newborns lose > 7% of their birthweight, some babies will continue breastfeeding and others will be finally switched to formula.

On the other hand, our findings expand on the association between milder degrees of birthweight loss and breastfeeding continuation. We did not find a difference of exclusive breastfeeding discontinuation at the more extreme end of birthweight loss, but we show that newborns whose birthweight loss at discharge was below the median were more likely to discontinue exclusive breastfeeding in the medium term. Our results are in line with Flaherman et al. findings [24], they have reported that duration of exclusive breastfeeding through 1 month was less likely among newborns whose discharge birthweight loss was >50th percentile, and we find the same by days 15, 30 and 100. It was expected that birthweight loss would be associated with decreased rate of exclusive breastfeeding in the very short term. In contrast, this was not true by day 7 but it was true thereafter. The underlying assumption is that birthweight loss trajectory is a marker of suboptimal breastfeeding efficiency in the medium term but not a trigger for imminent formula supplementation.

Finally, we found in our study that known predictors of breastfeeding cessation such as sex of child, parity, impaired glucose tolerance, and mode of delivery, were associated with breastfeeding cessation. Accordingly, the literature reports that sex of child impacts breastfeeding duration in India, Australia, Scandinavia, Latin America, and the US. It varies from country to country. Within the US, Hispanic women have lower odds of breastfeeding duration if they have sons compared to Hispanic women who have daughters [25]. The same applies to Brazil [26]. Also, Kenyan boys are more likely to be introduced to complementary feeding early compared with girls [27]. Conversely, Indian boys are breastfed more than girls, yet having few or no older brothers results in earlier weaning of daughters [28]. Our study shows that parity < 1 is an independent predictor of breastfeeding cessation from day 7 to day 100 of life. Previous studies show that breastfeeding duration increases with increasing parity [29, 30], which might be related to previous breastfeeding experiences. Cesarean section is a recognized risk factor for breastfeeding initiation failure. However, there is not absolute agreement on this point, the UK 2005 Infant Feeding Survey found that mothers were equally likely to be breastfeeding at 1 week regardless of delivery methods [31]. We report that cesarean section is significantly associated with breastfeeding cessation by weeks 1 and 2 of life, but our previous research found that mode of delivery was not associated with breastfeeding cessation [20], we do not have an explanation for this. Our findings add to the evidence that a woman’s impaired glucose intolerance adversely affect her lactation outcomes, this important association may account for some of the association with weight loss and exclusive breastfeeding outcomes. It is well established that overt gestational diabetes is associated with significantly increased risks of adverse breastfeeding outcomes [32]. In addition, emerging data suggest that lesser degrees of hyperglycemia also increase offspring risks, including early breastfeeding cessation [18, 33]. It has been shown that insulin-sensitive gene expression tends to be upregulated during the lactation cycle, that is why insulin plays a direct role in lactation [34].

### Limitations

First, data for patients’ characteristics were collected retrospectively in addition to routinely; therefore, lack of accuracy in this area is an expected shortcoming of the survey. Conversely, lack of accuracy of breastfeeding duration is not expected because these data were collected prospectively. Second, we have no detailed data on the many possible reasons for cessation of exclusive breastfeeding. We were not able to adjust for exclusive breastfeeding intention. Third, another limitation is that data are not available at the time of maximum weight loss, but reliability of our results is corroborated by the fact that the reported median weight loss of 6% corresponded to the 5.95% mean weight loss reported in a large birth cohort of term, healthy, exclusively breastfed neonates from a breastfeeding friendly birth center [9]. Finally, the studied population came from general care pediatric clinics in a middle-class neighborhood in Spain, which may not be representative of the general population. The data should be verified in a larger study encompassing more centers. We hope this study will help debunk some of the myths surrounding the rush for supplementation in cases of extreme weight loss.

## Conclusions

Our study asserts that high birthweight loss is associated with a higher risk of medium term cessation of exclusive breastfeeding, but we did not identify cut-off values to be used as predictors for the initiation of supplementary feeding. This research question remains unanswered. Similar to the criteria for different effective neonatal health interventions, the best cut-off value may vary widely according to child and mother clinical findings.

## Abbreviations

1-hOGTT`: 1-h 50-g-oral glucose tolerance test
3-hOGTT: 3-h 100-g-oral glucose tolerance test
VD: vaginal delivery
